# Assessing effects of reopening policies on COVID-19 pandemic in Texas with a data-driven transmission model

**DOI:** 10.1016/j.idm.2021.02.001

**Published:** 2021-02-23

**Authors:** Duo Yu, Gen Zhu, Xueying Wang, Chenguang Zhang, Babak Soltanalizadeh, Xia Wang, Sanyi Tang, Hulin Wu

**Affiliations:** a Department of Biostatistics and Data Science, The University of Texas Health Science Center at Houston, Houston, USA; b School of Mathematics and Information Science, Shaanxi Normal University, Xi’an, PR China

**Keywords:** COVID-19 pandemic, SEIR model, Texas state, Reopening business, Infectious disease transmission

## Abstract

While the Coronavirus Disease 2019 (COVID-19) pandemic continues to threaten public health and safety, every state has strategically reopened the business in the United States. It is urgent to evaluate the effect of reopening policies on the COVID-19 pandemic to help with the decision-making on the control measures and medical resource allocations. In this study, a novel SEIR model was developed to evaluate the effect of reopening policies based on the real-world reported COVID-19 data in Texas. The earlier reported data before the reopening were used to develop the SEIR model; data after the reopening were used for evaluation. The simulation results show that if continuing the “stay-at-home order” without reopening the business, the COVID-19 pandemic could end in December 2020 in Texas. On the other hand, the pandemic could be controlled similarly as the case of no-reopening only if the contact rate was low and additional high magnitude of control measures could be implemented. If the control measures are only slightly enhanced after reopening, it could flatten the curve of the COVID-19 epidemic with reduced numbers of infections and deaths, but it might make the epidemic last longer. Based on the reported data up to July 2020 in Texas, the real-world epidemic pattern is between the cases of the low and high magnitude of control measures with a medium risk of contact rate after reopening. In this case, the pandemic might last until summer 2021 to February 2022 with a total of 4–10 million infected cases and 20,080–58,604 deaths.

## Introduction

In early December 2019, the first Coronavirus Disease 2019 (COVID-19) case was identified in Wuhan, China, which was caused by severe acute respiratory syndrome coronavirus 2 (SARS-CoV-2) (Guan et al., 2020). COVID-19 has rapidly spread to most of the cities in China since then, and eventually caused the pandemic in the world. The outbreak of COVID-19 was declared as a pandemic by the World Health Organization on March 11th (World Health Organization, 2020). As of May 16, 2020, a total of 4,434,653 confirmed cases and 302,169 deaths had been reported globally (WHO Coronavirus Disease (COVID-19) Dashboard, 2020). Among all the counties and regions, the United States has been the most worrisome country which has the most confirmed cases and deaths. The number of cases and deaths reached to more than 7 million and 200, 000 respectively in October 2020.

Due to the absence of vaccine and treatment, the prevention and control of COVID-19 have mainly relied on behavioral prevention measures, which include keeping social distance, wearing masks, stay-at-home order, and closure of nonessential business (Yu et al., 2017). In the United States, most of the states had declared a state of emergency and issued a stay-at-home order. Although many people have been prevented from infection through social distancing strategies, tens of millions of unemployment claims have been filed during COVID-19 outbreak in the United States (U.S. Bureau of Labor Statistics). As more and more people want to return to work, slowing down the spread of SARS-CoV-2 becomes more challenging.

Recently, the stay-at-home order has been lifted across the country and some business restrictions have also been relaxed (CNN, 2020). Since the pandemic has not been completely controlled, the daily new confirmed cases are continually reported, it is urgent to evaluate the effect of the reopening policies on the COVID-19 pandemic to help manage the future medical resources and control measures. It has been observed that the number of daily confirmed cases is approaching another peak after the reopening policies are released in several states, including Texas, Alabama and South Dakota (COVID-19 tracking project (The Atlantic, The Covid Tracking Project, 2020)). For example, between April 10th and 30th, 2020, the numbers of daily confirmed cases were never greater than 1000 in Texas (Texas DSHS, Texas COVID-19, 20202), but the average number of daily confirmed cases between April 30th and May 16th, 2020 was 1173, in particular, the number of daily confirmed cases approached 1801 on May 16th, 2020 after a reopening policy was implemented (Texas DSHS, Texas COVID-19 Data, 2020). The increase in number of daily confirmed cases after reopening was likely due to the increased contact rate of susceptible people with infected cases. But it is not clear whether there is a second wave of outbreak and what the total number of cases will reach after reopening. It is crucial to understand the effect of reopening policies on the pandemic to prevent more infections. The pandemic situation in different states and counties are different and the local governments make their own decisions on when and how to reopen the business. In this study, we mainly focused on evaluating the reopening policies in Texas, although the methodologies in this paper are applicable to other states or regions in general.

As for disease transmission, different mathematical models and statistical methods have been applied to predict the future trend, which include multivariate linear regression (Thomson et al., 2006), grey forecasting method (Wang, 2018), time series techniques and neural networks (Liu et al., 2016) and the susceptible/exposed/infective/recovered (SEIR) model (Sanyi et al., 2020; Xia et al., 2020; Yu et al., 2017) among others. In particular, the SEIR model is the most popular mathematical model which have been employed to study the COVID-19 pandemic since its outbreak in China (Afonso et al., 2020; Das et al., 2020; He et al., 2020; López & Rodo, 2020; Wu et al., 2020; Yang et al., 2020; Zhan et al., 2020; Zhao et al., 2020). In this study, we proposed a modified SEIR model to study the COVID-19 epidemic in Texas. Using the proposed model, we estimated the COVID-19 epidemic parameters based on the data of reported infected cases and deaths in Texas, and predicted the future epidemics of COVID-19 under different reopening policies. The results of this study can help the local governments make decisions on pandemic control and future reopening policies.

### SEIR model and estimation method

We assume that the overall population can be divided into nine compartments: susceptible individuals (S), exposed individuals who are not quarantined (E), exposed and quarantined individuals ($E_{q}$), infected individuals with symptoms ($I_{s}$), infected individuals with no symptoms ($I_{a}$), confirmed cases who are quarantined at home ($H_{1}$), confirmed cases who are hospitalized ($H_{2}$), recovered individuals (R), and individuals who died (D). A SEIR model is modified to capture the details of prevention and control measures based on the model in the literature (Sanyi et al., 2020; Wang et al., 2020b, Wang et al., 2020a; Wang et al., 2020b, Wang et al., 2020a). The proposed SEIR model is written as follows:

(1) $$S^{\text{′}}=\lambda E_{q}+\mu E-\frac{c_{1}\left(t\right)SI_{s}}{N}-\frac{c_{2}\left(t\right)SI_{a}}{N}$$

(2) $$E^{\text{′}}=\frac{c_{1}\left(t\right)SI_{s}}{N}+\frac{c_{2}\left(t\right)SI_{a}}{N}-q\left(t\right)E-\sigma E-\mu E$$

(3) $$E_{q}^{\prime }=\text{q}\left(\text{t}\right)\text{E}-\;\lambda E_{q}-\;\beta E_{q}$$

(4) $$I_{s}^{\text{′}}=\rho \sigma E+\omega_{2}\gamma_{a}I_{a}-\delta_{s}\left(t\right)I_{s}\left(t\right)$$

(5) $$I_{a}^{\text{′}}=\left(1-\rho \right)\sigma E-\gamma_{a}I_{a}-\delta_{a}\left(t\right)I_{a}\left(t\right)$$

(6) $$H_{1}^{\text{′}}=\omega_{5}\beta E_{q}+\delta_{a}\left(t\right)I_{a}+\omega_{1}\delta_{s}\left(t\right)I_{s}-\gamma_{1}H_{1}$$

(7) $$H_{2}^{\text{′}}=\left(1-\omega_{5}\right)\beta E_{q}+\left(1-\omega_{1}\right)\delta_{s}\left(t\right)I_{s}+\omega_{3}\gamma_{1}H_{1}-\gamma_{2}H_{2}$$

(8) $$R^{\text{′}}=\left(1-\omega_{2}\right)\gamma_{a}I_{a}+\left(1-\omega_{3}\right)\gamma_{1}H_{1}+\omega_{4}\gamma_{2}H_{2}$$

(9) $$D^{\text{′}}=\left(1-\omega_{4}\right)\gamma_{2}H_{2}$$

**Fig. 1:**
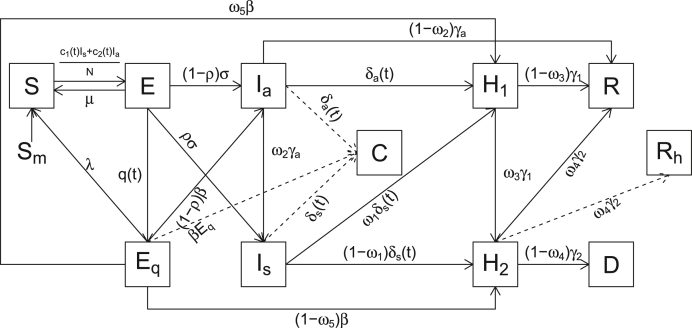
The proposed SEIR model diagram.

This model assumes that the total number of the population keeps constant, i.e., no migration is considered. The individuals in the infected components, $I_{s}$ and $I_{a}$, are the only groups to infect the susceptible people. The transmission dynamic is modelled as follows (see the flowchart in Fig. 1). Susceptible individuals, S, could become the exposed individuals who were not quarantined, E, after they were exposed to infected individuals $I_{s}$ and $I_{a}$, see equation (1) where $c_{1}\left(t\right)$ and $c_{2}\left(t\right)$ denote the contact rate of infected people with symptoms and without symptoms, respectively. The exposed individuals (E) further could move to the quarantined, infected and susceptible components with different rates, respectively. Specifically, they could move to exposed and quarantined component $E_{q}$ with a quarantine rate q(t), to infected components, $I_{s}$ and $I_{a}$, with an infection rate σ, and the susceptible component S with a rate μ. Individuals in component $E_{q}$ could become infectious with an infection rate β (Mizumoto et al., 2020). Note that the compartment consisting of exposed individuals (E) is different from the traditional SEIR model (Aron & Schwartz, 1984; Wang et al., 2020b, Wang et al., 2020a; Wang et al., 2020b, Wang et al., 2020a), where the exposed individuals are those infected patients but not yet infectious. Here we define the exposed individuals as those who literally contacted with the infected individuals and have not been infected yet. The infected patients with symptoms were detected to become confirmed cases and moved to confirmed and quarantined component $H_{1}$ with a rate $\delta_{s}\left(t\right)$. The detection rate for infected people with no symptoms is $\delta_{a}\left(t\right).$ Individuals who were infected without symptoms could move to the symptomatic infected component $I_{s}$ with a rate $\omega_{2}\gamma_{a}$ or recovered component R with a rate$\left(1-\omega_{2}\right)\gamma_{a}.$ Individuals who were confirmed cases and quarantined at home could move to hospitalized component $H_{2}$ and recovered component R with a rate $\gamma_{1}$. Hospitalized patients could move to recovered and death component, R and D, with a rate $\gamma_{2}$. The recovered cases cannot be infected again. In this model, $\omega_{1}$ denotes the proportion of confirmed infected people with mild symptoms (no need for hospitalization) (Covid & Team, 2020); $\omega_{3}$ denotes the probability of confirmed cases quarantined at home becoming to be hospitalized; $\omega_{4}$ denotes the proportion of hospitalized COVID-19 patients who recovered (Wang et al., 2020b, Wang et al., 2020a; Wang et al., 2020b, Wang et al., 2020a); $\omega_{5}$ is the proportion of the patients who were confirmed with infection and quarantined at home.

We assume some parameters to be time varying to reflect the real world change due to policy changes, such as the contact rate, detection rate, quarantine rate and death rate. Specifically, the contact rate of infected people with symptoms $c_{1}\left(t\right)$ is modelled as

(10) $$\left\{ \begin{array}{l} c_{1}\left(t\right)=c_{10},\;t\leq T_{1c};\\ c_{1}\left(t\right)=\left(c_{10}-c_{1b}\right)e^{-r_{1}\left(t-T_{1c}\right)}+c_{1b},\;t>T_{1c}\; \end{array}\right.$$

where $c_{10}$ is the baseline contact rate of the infected people with symptoms, $c_{1b}$is the minimum contact rate of infected people with symptoms under control strategies, $c_{1b}<c_{10}$, and $r_{1}$ denotes how an exponential decrease in the contact rate is achieved. Critical time $T_{1c}$ denotes the timing of social distancing strategies implemented on March 19th when Texas declared of state disaster and prohibited 10+ person of gathering (Office of Texas Governor, 2020). Similarly, the contact rate of infected people without symptoms $c_{2}\left(t\right)$ is modelled as

(11) $$\left\{ \begin{array}{l} c_{2}\left(t\right)=c_{20},\;t\leq T_{1c};\\ c_{2}\left(t\right)=\left(c_{20}-c_{2b}\right)e^{-r_{1}\left(t-T_{1c}\right)}+c_{2b},\;t>T_{1c}\; \end{array}\right.$$

where $c_{20}$ is the baseline contact rate of infected people without symptoms, $c_{2b}$ is the minimum contact rate of infected people without symptoms under control strategies, $c_{2b}<c_{20}$, and $\;r_{2}$ denotes how an exponential decrease in the contact rate of the infected people without symptoms is achieved. The transition rate of exposed people from non-quarantined state to quarantined, q(t), is

(12) $$\left\{ \begin{array}{l} q\left(t\right)=q_{0},t\leq T_{2c};\\ q\left(t\right)=\left(q_{0}-q_{m}\right)e^{-r_{3}\left(t-T_{2c}\right)}+q_{m},t>T_{2c} \end{array}\right.$$

where $q_{0}$ denotes the baseline quarantine rate, $q_{m}$ denotes the maximum quarantine rate, $q_{m}>q_{0}$, $r_{3}$ denotes the parameter of exponential increase in the quarantine rate, and $T_{2c}$ denotes the critical timing of enhanced quarantine strategies on April 2nd, 2020 when Texas declared stay-at-home order (Office of the Texas Covernor, 2020). The detection rate for infected people with symptoms was modelled as $\delta_{s}\left(t\right)=\phi_{s}\left(t\right)s$, where

(13) $$\left\{ \begin{array}{l} \frac{1}{\phi_{s}\left(t\right)}=\frac{1}{\phi_{s0}},t\leq T_{3c};\\ \frac{1}{\phi_{s}\left(t\right)}=\;\left(\frac{1}{\phi_{s0}}-\frac{1}{\phi_{sf}}\right)e^{-r_{4}\left(t-T_{3c}\right)}+\frac{1}{\phi_{sf}},t>T_{3c} \end{array}\right.$$

$\varphi_{s}\left(t\right)$ is the testing rate of infected people with symptoms (Heneghan et al., 2020), $\varphi_{s0}$ is the baseline of the test rate of infected people with symptoms, $\phi_{sf}$ is the maximum of the test rate of infected people with symptoms, and s denotes the sensitivity of the testing kit. Critical time $T_{3c}$ denotes the timing of enhanced detecting rate for people with symptoms on March 17th, 2020 when the first drive-thru testing site was open for public in Texas (O.o.T. Governor, 2020). The detection rate for infected people without symptoms was modelled as $\delta_{a}\left(t\right)=\phi_{a}\left(t\right)s$, where

(14) $$\left\{ \begin{array}{l} \frac{1}{\phi_{a}\left(t\right)}=\frac{1}{\phi_{a0}},t\leq T_{3c};\\ \frac{1}{\phi_{a}\left(t\right)}=\;\left(\frac{1}{\phi_{a0}}-\frac{1}{\phi_{af}}\right)e^{-r_{5}\left(t-T_{3c}\right)}+\frac{1}{\phi_{af}},t>T_{3c} \end{array}\right.$$

where $\phi_{a}\left(t\right)$ is the testing rate of infected people without symptoms, $\varphi_{a0}$ is baseline of the test rate of infected people without symptoms, $\phi_{af}$ is the maximum of the test rate of infected people without symptoms, and s is the sensitivity of the testing kit. Critical time $T_{3c}$ denotes the timing of enhanced detecting rate for people without symptoms (O.o.T. Governor, 2020). The proportion of hospitalized COVID-19 patients who recovered (Richardsonet al., 2020; Zhouet al., 2020), is modelled as

(15) $$\omega_{4}\left(t\right)=\left(\omega_{40}-\omega_{4b}\right)e^{-r_{6}t}+\omega_{40}$$

where $\omega_{40}$ denotes the baseline recovery proportion, $\omega_{4b}$ denotes the maximum recovery proportion, $\omega_{4b}>\omega_{40}$ , and $r_{6}$ denotes the parameter of exponential increase in the recovery proportion.

For model fitting and parameter estimation, we used the COVID-19 data collected from the Texas Department of State Health Services (Texas DSHS, 2020; The Atlantic, The Covid Tracking Project, 2020). Particularly, we used the number of cumulative confirmed cases and the number of cumulative deaths from hospitalized COVID-19 patients from March 4th to April 28th, 2020, for parameter estimation. The source data for the number of hospitalizations and recovered patients were not directly observed, instead they were estimated, which may not be reliable and were not used in our model fitting. We assume the observed data model as,

(16) $$\begin{array}{l} Y_{c}\left(t\right)=C\left(t,\;\theta \right)+\varepsilon_{1}\\ Y_{d}\left(t\right)=D\left(t,\theta \right)+\varepsilon_{2} \end{array}$$

where *Y*_*c*_*(t)* and *Y*_*d*_*(t)* denote the reported numbers of cumulative COVID-19 confirmed cases and deaths in Texas, respectively. The measurement errors, $\varepsilon_{1}$ and $\varepsilon_{2}$, are assumed as normal distribution with mean 0 and variance σ^2^. C(t,θ) is the predicted number of cumulative confirmed cases by the SEIR model which was estimated by the following differential equation,

(17) $$\frac{d}{\partial t}C\left(t,\;\theta \right)=\delta_{a}\left(\text{t}\right)I_{a}+\delta_{s}\left(\text{t}\right)I_{s}+\;\beta E_{q}$$

D(t,θ) is the predicted number of cumulative deaths by the SEIR model, which was calculated through equation (9). θ denotes the model parameters. The nonlinear least squares (NLS) estimation method can be used to estimate the SEIR model parameters (Cao et al., 2012). The NLS objective function for our SEIR model is

(18) $$L\left(\theta ;Y_{c}\left(t\right),Y_{d}\left(t\right)\right)=\overset{T}{\underset{t=0}{\sum }}\left[{\left(Y_{c}\left(t\right)-C\left(t\right)\right)}^{2}+{\left(Y_{d}\left(t\right)-D\left(t\right)\right)}^{2}\right].$$

To alleviate the model identification problem (Miao et al., 2011), we fixed some parameters according to literature (see Table 1). In addition, all the unknown parameters were constrained with pre-defined lower and upper bounds (see Table 2). The interior-point method was used to optimize the loss function (18), and implemented with MATLAB.

**Table 1:** Values of the fixed parameters based on literature.

Fixed Parameters	Definition	Values
N	The population size	28,995,881
s	Sensitivity of the test for infected people	0.8 (Ai et al., 2020; Wang et al., 2020)
$\omega_{1}$	Probability of confirmed cases to be quarantined at home	0.81 (C.f.D. 2020; T.C. COVID, and R. Team, 2020)
S (0)	The initial value of the number of the susceptible individuals	28,995,881
$E_{q}\left(0\right)$	The initial value of the number of the exposed and quarantined individuals	0
$H_{1}\left(0\right)$	The initial value of the number of the patients who are confirmed cases quarantined at home	0
$H_{2}\left(0\right)$	The initial value of the number of the confirmed cases and hospitalized individuals	1
R(0)	The initial value of the number of recovered individuals	0
D(0)	The initial value of the number of the deaths	0

**Table 2:** Parameter estimates based on the least square with constraints.

Parameters		Definition	Initial value	Lower bound	Upper bound	Estimated value
E(0)		Initial value of exposed but not quarantined population	200	50	1000	224.03
$I_{s}\left(0\right)$		Initial value of infected patients with symptoms	20	10	150	149.74
$I_{a}\left(0\right)$		Initial value of infected patients with no symptoms	15	10	200	14.616
λ		Transition rate from exposed and quarantined to susceptible	0.05	0.01	0.5	0.4975
μ		Transition rate from exposed and not quarantined to susceptible	0.1	0.01	0.9	0.6328
$c_{1}\left(t\right)$	$c_{10}$	Baseline contact rate of infected people with symptoms	1.5	0.5	5	1.8326
	$c_{1b}$	Minimum contact rate of infected people with symptoms under control strategies	0.5	0.005	2	0.8401
	$r_{1}$	Denotes how an exponential decrease in the contact rate of infected people with symptoms is achieved	0.15	0.005	0.4	0.0742
$c_{2}\left(t\right)$	$c_{20}$	Baseline contact rate of infected people without symptoms	3	0.5	10	3.2825
	$c_{2b}$	Minimum contact rate of infected people without symptoms under control strategies	1	0.005	2	0.9444
	$r_{2}$	Denotes how an exponential decrease in the contact rate of infected people without symptoms is achieved	0.1	0.001	0.4	0.0010
q(t)	$q_{0}$	Baseline quarantine rate	0.1	0.01	0.2	0.0504
	$q_{m}$	Maximum quarantine rate under control strategies	0.05	0.04	0.1	0.0536
	$r_{3}$	Denotes how an exponential increase in the quarantined rate is achieved	0.15	0.05	0.5	0.4998
σ		Transition rate of exposed and not quarantined to infected	0.05	0.01	0.2	0.0722
β		Infection rate of exposed quarantined patient	0.05	0.01	0.2	0.0100
ρ		Probability of having symptoms for an infected patient	0.25	0.1	0.6	0.4835
$\gamma_{a}$		Transition rate from infected patients with no symptoms to patients with symptoms or recovered	0.1	0.01	0.9	0.6361
$\delta_{s}\left(t\right)$	$\phi_{s0}$	Baseline of the test rate of infected people with symptoms	0.0333	0.01	0.1429	0.0100
	$\phi_{sf}$	Maximum of the test rate of infected people with symptoms	0.3333	0.0714	0.5	0.1197
	$r_{4}$	Denotes how an exponential increase in the test rate of infected people with symptoms is achieved	0.15	0.001	0.4	0.2803
$\delta_{a}\left(t\right)$	$\phi_{a0}$	Baseline of the test rate of infected people without symptoms	0.0167	0.01	0.0714	0.0435
	$\phi_{af}$	Maximum of the test rate of infected people without symptoms	0.0714	0.0333	0.5	0.0334
	$r_{5}$	Denotes how an exponential decrease in the test rate of infected people without symptoms is achieved	0.05	0.0001	0.15	0.1282
$\gamma_{1}$		Transition rate from confirmed cases quarantined at home to hospital or recovered	0.07	0.01	0.1429	0.1423
$\gamma_{2}$		Transition rate from confirmed cases quarantined at hospital to recovered or death	0.05	0.01	0.1429	0.1428
$\omega_{2}$		Probability of infected patients without symptoms transit to have symptoms	0.1	0.05	0.5	0.3953
$\omega_{3}$		Probability of confirmed cases quarantined at home transit to be hospitalized	0.1	0.1	0.5	0.4998
$\omega_{5}$		The proportion of confirmed patients who are quarantined at home	0.85	0.7	0.9	0.7045
$\omega_{4}\left(t\right)$	$\omega_{40}$	Baseline of the recovery rate of confirmed and hospitalized cases	0.85	0.85	0.99	0.8500
	$\omega_{4b}$	Maximum of the recovery rate of confirmed and hospitalized cases	0.92	0.92	0.96	0.9599
	$r_{6}$	Denotes how an exponential increase in the recovery rate of confirmed and hospitalized cases	0.08	0.01	0.1	0.0197

To evaluate the effect of fixed parameters and initial values on the estimation results, sensitivity analyses were performed. For the fixed parameters $\omega_{1}$ and s, we selected 6 values around the default value for each parameter in Table 1. Then, a total of 36 different combinations of the fixed parameters were used to refit the model. For the initial values of parameters, each time we randomly selected one value around the estimated value for each parameter as the new initial value. Then we refitted the model using the generated new initial values. After repeating above steps for 800 times, we obtained the estimation results from the 800 different initial value sets. The distribution of the objective function values can be used to evaluate whether the solution is optimal. The comparison of prediction results under different parameter settings is another way to assess the influence of parameters on modelling results. If the prediction results are close, we can conclude that our model prediction is robust for the variation of parameters.

Once the model is established and model parameters are estimated, we simulated the COVID-19 epidemic in Texas under the assumption of different reopening policies. The simulation focused on the effect of different reopening magnitudes on the COVID-19 epidemics in Texas. In the simulations, the timing of actual Texas reopening policies was used. Texas has implemented three phases of reopening which were effective on May 1st, May 18th and June 3rd, 2020 respectively (KHOU, 2020). Due to the increase in COVID-19 cases and hospitalizations, Texas governor announced the temporary pause of additional reopening on June 25th. We quantified the effect of reopening policies by the fold-change of the contact rate, quarantine rate, and detection rate from those on April 30th, the day before the phase one reopening effective day. The simulation mainly focused on three different levels of risk of reopening which were quantified by the change of the contact rate. Given a certain risk of reopening, we simulated three different magnitudes of control strategies which were quantified by the quarantine and detection rates.

### Estimation and policy simulation results

**Fig. 2:**
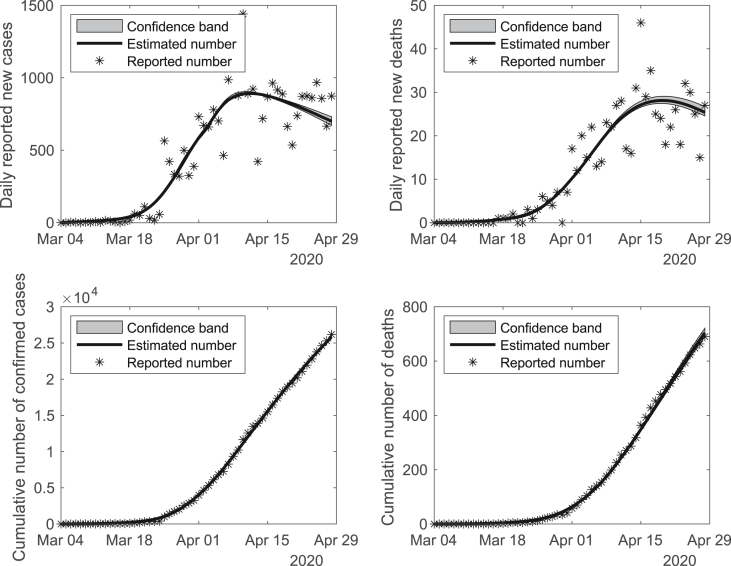
Model fitting with the estimated parameters based on the observed cumulative number of reported cases and deaths in Texas from March 4th to April 28th, 2020.

According to the Texas Department of State Health Services (DSHS) (Texas DSHS, 2020), the first confirmed COVID-19 case in Texas was documented on March 4th, 2020. The patient was a man in his 70s who travelled aboard and returned to Texas with symptoms, then was hospitalized immediately after diagnosis. Therefore, we assumed the initial value of the number of hospitalized individuals as 1 on March 4th, 2020. The initial value of the number of susceptible individuals was assumed as the whole Texas population (N = 28,995,881). In addition, we fixed some of the model parameters based on literature in order to alleviate the model identifiability problem (see Table 1). With the constrained least squares (LS) method, the rest of the unknown parameters in the ODE system were estimated based on reported number of confirmed cases and deaths in Texas which are shown at Table 2. The proposed model fits the observed COVID-19 data from Match 4th to April 28th, 2020 very well (see Fig. 2). To further valid the model fitting, we performed sensitivity analyses against both fixed parameters and the initial values of parameters (see Figures S3–S10 in the supplemental material). It shows that the model fitting and prediction results are quite robust to the variations of fixed parameters and initial values. We also notice that our final parameter estimates may not be the best solution in terms of the objective function in the sensitivity analyses, but they are very close to the optimal point.

We modelled the reopening policy with three levels of risk, i.e., low, medium and high risks that are quantified by the contact rate with infected individuals without symptoms (we assume that the effective contact rate with infected individuals with symptoms could be ignored). The changes of quarantine rate and detection rates were used to quantify the control measures under different reopening policies. The first phase of reopening in Texas was announced on April 27th and effective on May 1st, 2020. Thus, the estimates of the time-varying parameters, the contact rate, quarantine rate and detection rates on April 30th, 2020 were used as the baseline values for the changes of reopening policies. Based on the proposed SEIR model, the estimated baseline contact rate for patients without symptoms was 4.088; the baseline quarantine rate was 0.104; the baseline detection rate for patients with symptoms was 0.104; and the baseline detection rate for patients without symptoms was 0.061. We quantified the “low-risk” reopening policy as the contact rate increased by 2 times on May 1st, 3 times on May 18th, 4 times on June 3rd, then reduced to 3 times on June 25th, 2020 due to the reopening pause. The medium and high-risk reopening policies were similarly defined but with a larger magnitude of change in the contact rate after reopening. For each level of the reopening risk, we simulated three scenarios of the control measures based on the quarantine rate and detection rates, i.e., 1) no change of control measures; 2) low magnitude of control measures where the quarantine and detection rates increased by 1.5 folds; and 3) high magnitude of control measures where the quarantine and detection rates increased by 2 folds. The detailed simulation design for the Texas reopening policy are summarized in Table 3. The daily and cumulative numbers of confirmed cases, deaths, infected and hospitalized patients were evaluated under different simulation scenarios between May 1st and September 30th, 2020. The reported daily and cumulative confirmed COVID-19 cases and deaths between May 1st and July 31st, 2020 were used for comparisons with the simulated results.

**Table 3:** Texas reopening policy scenarios: the change of contact rate, quarantine rate, and detection rates were compared with those on April 30th.

Scenarios		Change of contact rate (fold-change)					Change of quarantine rate (fold-change)	Change of detection rates (fold-change)
Contact risk	Control measures	May 1st	May 18th	June 3rd		June 25th		
$S_{0}$: no reopening		–	–	–		–	–	–
Low-risk	$S_{11}$: no change	2	3		4	3	–	–
	$S_{12}$: low control	2	3		4	3	1.5	1.5
	$S_{13}$: high control	2	3		4	3	2	2
Medium-risk	$S_{21}$: no change	3	4		5	4	–	–
	$S_{22}$: low control	3	4		5	4	1.5	1.5
	$S_{23}$: high control	3	4		5	4	2	2
High-risk	$S_{31}$: no change	4	5		6	5	–	–
	$S_{32}$: low control	4	5		6	5	1.5	1.5
	$S_{33}$: high control	4	5		6	5	2	2

**Fig. 3:**
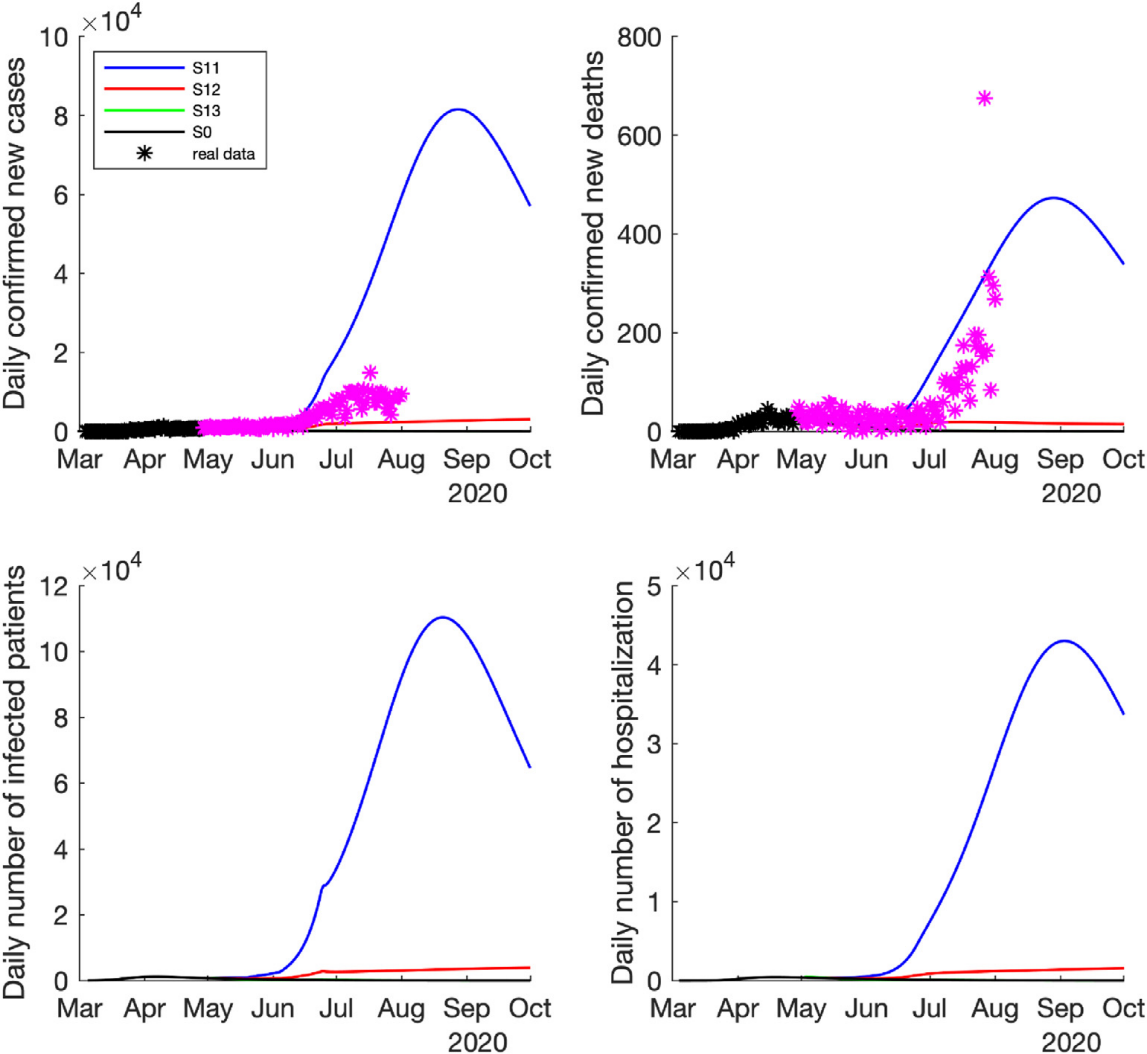
Predicted daily number of new confirmed cases, deaths, infected cases and hospitalized cases if the low-risk reopening policy was implemented, i.e., the contact rate increased by 2 times on May 1st, increased by 3 times on May 18th, increased by 4 times on June 3rd, and reduce to 3 times after June 25th. The time span is between March 4th and October 1st, 2020. The black ∗ denotes the reported data used for model fitting and purple ∗ denotes the reported data after model fitting.

**Fig. 4:**
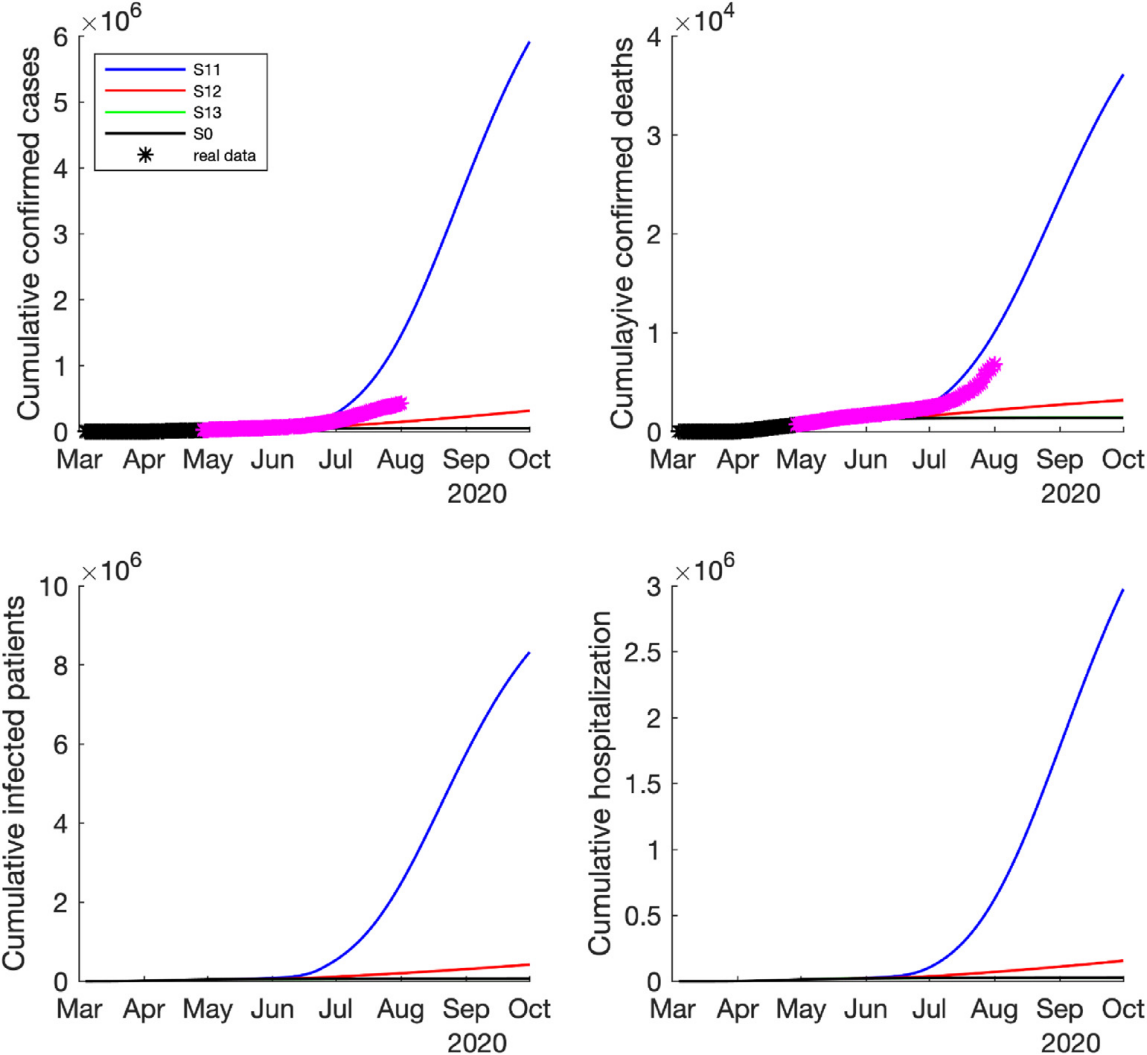
Predicted cumulative number of new confirmed cases, deaths, infected cases and hospitalized cases if the low-risk reopening policy was implemented, i.e., the contact rate increased by 2 times on May 1st, increased by 3 times on May 18th, increased by 4 times on June 3rd, and reduce to 3 times after June 25th. The time span is between March 4th and October 1st, 2020. The black ∗ denotes the reported data used for model fitting and purple ∗ denotes the reported data after model fitting.

Under the low-risk reopening policy (i.e., the effective contact rate only increased by 2–4 folds after reopening), if the high magnitude of control measures were implemented (i.e., the detection rates and quarantine rate were enhanced by 2-folds), the pandemic would be well controlled, which was similar to the scenario where no reopening policy was applied, see the epidemic curves of $S_{13}$ and $S_{0}$ in Fig. 3, Fig. 4. However, if only a low magnitude of control measures (the detection rates and quarantine rate were enhanced by 1.5-folds), the pandemic would slowly become worse (see the curves of $S_{12}$ in Fig. 3, Fig. 4). The worst case is that, if no additional control measures were adopted after reopening (no change in the detection rates and quarantine rate), the number of infected cases, hospitalizations and deaths could be rapidly increased with a peak around the late August and early September 2020, see the curves of scenario $S_{11}$ in Fig. 3, Fig. 4.

**Fig. 5:**
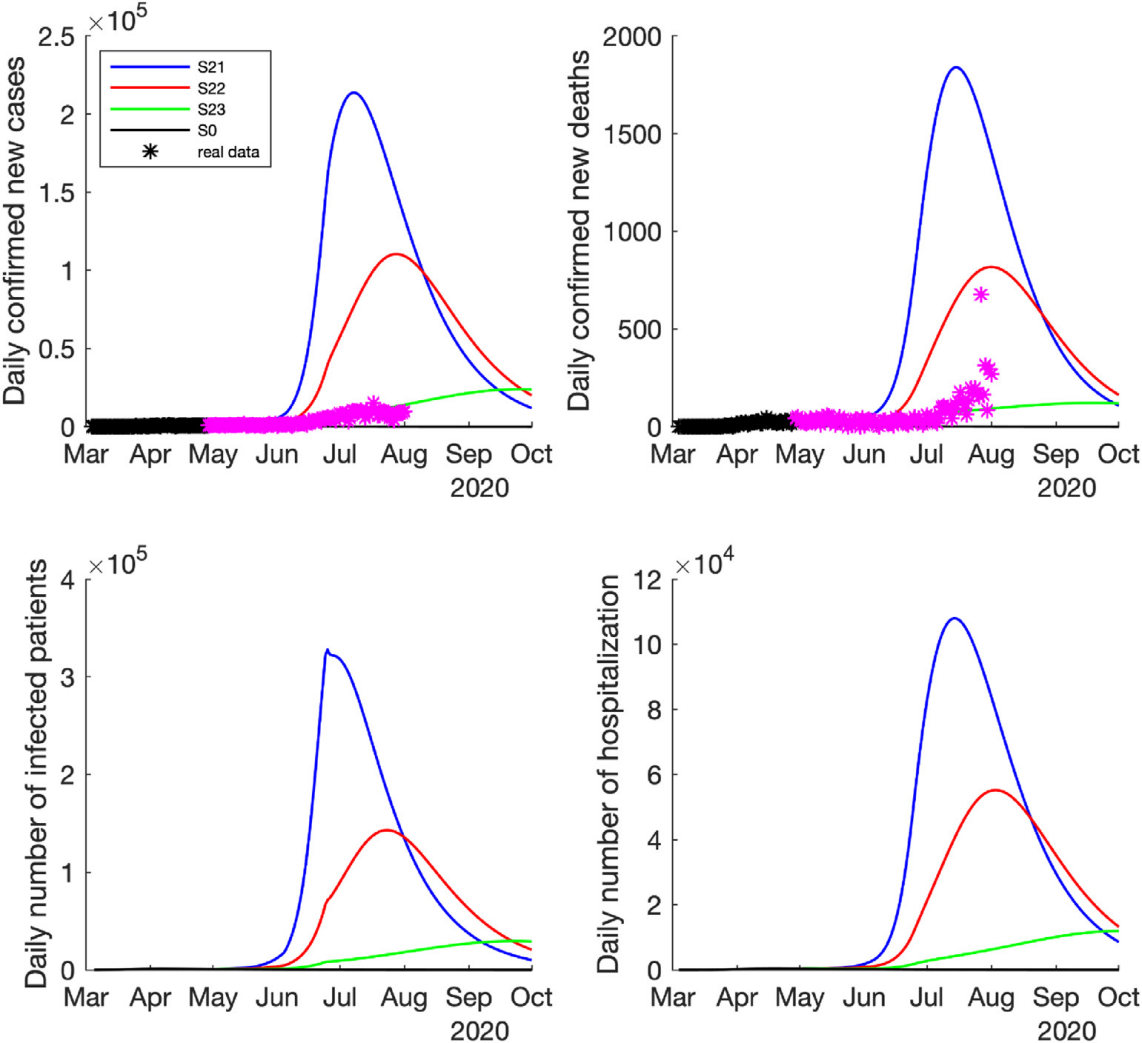
Predicted daily number of new confirmed cases, deaths, infected cases and hospitalized cases if the medium-risk reopening policy was implemented, i.e., the contact rate increased by 3 times on May 1st, increased by 4 times on May 18th, increased by 5 times on June 3rd, and reduce to 4 times after June 25th. The time span is between March 4th and October 1st, 2020. The black ∗ denotes the reported data used for model fitting and purple ∗ denotes the reported data after model fitting.

**Fig. 6:**
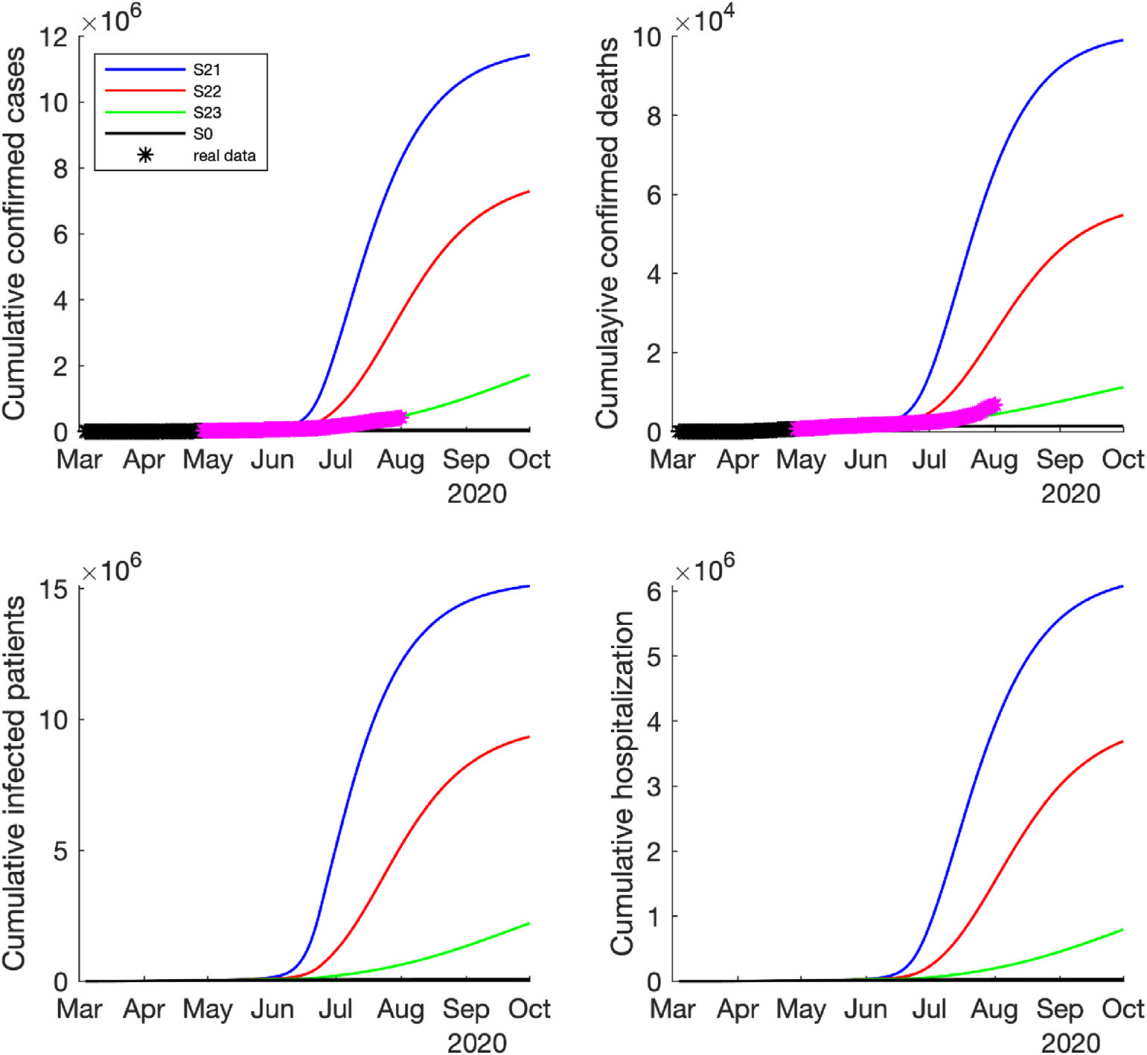
Predicted cumulative number of new confirmed cases, deaths, infected cases and hospitalized cases if the medium-risk reopening policy was implemented, i.e., the contact rate increased by 3 times on May 1st, increased by 4 times on May 18th, increased by 5 times on June 3rd, and reduce to 4 times after June 25th. The time span is between March 4th and October 1st, 2020. The black ∗ denotes the reported data used for model fitting and purple ∗ denotes the reported data after model fitting.

For the medium risk of reopening case (i.e., the effective contact rate only increased by 3–5 folds after reopening), all the simulation results show that the pandemic would resurge rapidly after reopening. Among the three cases with different magnitudes of control measures, the high magnitude of control measures (the detection rates and quarantine rate were enhanced by 2-folds) could delay the timing of next pandemic wave and reduce the wave magnitude significantly, i.e., the epidemic curve could be flattened (see the curve $S_{23}$ in Fig. 5, Fig. 6). However, if there were no additional control measures adopted after reopening in this case, the number of daily COVID-19 cases, hospitalizations and deaths could dramatically increase to the peak in early July 2020 (see the curve S_21_ in Fig. 5). If the low magnitude of control measures were implemented (the detection rates and quarantine rate were enhanced by 1.5-folds), the pandemic would start to get worse in the middle of June and reach to the peak later in August 2020, but the peak would be much lower than the case without additional control measures (see the curve S_22_ in Fig. 5, Fig. 6). Fortunately, the real-world reported confirmed new cases and deaths in Texas (stars in Fig. 5, Fig. 6) were between S_22_ and S_23_, which might indicate that the medium control measures might have been implemented (i.e., the detection rates and quarantine rate were enhanced between 1.5-folds and 2-folds).

On the contrary, for the high-risk reopening policy (i.e., the effective contact rate increased by 4–6 folds after reopening), our simulation results show that the COVID-19 epidemic could not be controlled by even the high magnitude of control measures, and the epidemic could rapidly move to a new wave and different control measures barely had any effect on the timing of next big wave, but only reduced the magnitude of the wave (see Figures S1–S2 in the supplemental material).

We also simulated the complete COVID-19 epidemic trajectories until the end of the epidemic in Texas for different scenarios as described above. The final results are summarized in Table 4. Based on the epidemic trajectory simulations, we observed that, if the stay-at-home order continued instead of reopening after May 2020, the pandemic would be under control ($S_{0}$) and end in December 2020, result in a total of 67,196 infections, 1394 deaths, and 27,582 hospitalizations in Texas. After the reopening policy was implemented, the pandemic could still be under control only if the reopening is a low-risk with a high magnitude of control measures adopted (2-folds increase in the detection rates and quarantine rate), see the curves of $S_{13}$ in Fig. 3, Fig. 4. In this case, the pandemic will end in November 2020 and the total number of infected cases, hospitalizations and deaths would be similar to the case without reopening (see Table 4). However, if no any control measures were enhanced after reopening, even under the low risk case, the pandemic might last for one more year, probably end in October 2021 with 49,651 in death toll and more than 37% of the Texas population infected. If only a low magnitude of control measures was implemented (1.5-folds increase in the detection rates and quarantine rate), the pandemic might last many years (until spring 2024), but with a smaller death toll, hospitalization and infection counts (see the case S_12_ in Table 4) compared to the case without additional control measures (the case S_11_ in Table 4).

**Table 4:** Effect of different reopening policies on COVID-19 pandemics in Texas.

Scenarios		Pandemic end date	Total Infections	Total deaths	Total hospitalizations
Contact risk	Control measures				
$S_{0}$: no reopening		11-Dec-2020	67,196	1394	27,582
Low-risk	$S_{11}$: no change	18-Oct-2021	10,783,692	49,651	4,414,398
	$S_{12}$: low control	01-Mar-2024	1,792,414	7566	733,011
	$S_{13}$: high control	12-Nov-2020	66,367	1417	27,229
Medium risk	$S_{21}$: no change	30-Apr-2021	15,325,653	101,305	6,273,655
	$S_{22}$: low control	04-Jun-2021	9,850,955	58,604	4,028,101
	$S_{23}$: high control	01-Feb-2022	4,336,288	20,080	1,770,218
High-risk	$S_{31}$: no change	28-Feb-2021	17,961,572	142,578	7,352,671
	$S_{32}$: low control	09-Feb-2021	14,069,211	112,202	5,752,920
	$S_{33}$: high control	10-Mar-2021	10,421,684	76,884	4,254,285

If the reopening policy was implemented on May 1st, 2020 with a medium risk of contact (i.e., the effective contact rate increased by 3–5 folds after reopening) and no additional control measures were implemented after reopening, COVID-19 pandemic might end in April 2021 with a significantly higher total death (101,305) and as many as more than 50% of Texas population could be infected. However, if the additional control measures were implemented (1.5 to 2-folds increase in the detection rates and quarantine rate), COVID-19 pandemic could last longer (end in June 2021 or February 2022), but the total number of deaths, hospitalizations and infected cases could be significantly reduced (see cases S_22_ and S_23_ in Table 4) compared to the case with no change of controls measures (S_21_). Particularly, the total deaths could be reduced by more than 40% if the control measures could increase by 1.5-folds and more than 80% if the control measures could increase by 2-folds. If the reopening policy was implemented on May 1st, 2020 with a high-risk of contact (the effective contact rate increased by 4–6 folds after reopening), COVID-19 pandemic might last for one year (end in spring 2021), but it would result in 10–17 million infections and as high as 142,578 deaths (if no additional control measures were adopted), which is a pandemic disaster.

## Conclusions and discussion

In this study, we developed a comprehensive SEIR model to capture the SARS-CoV-2 transmission based on which we assessed the effect of different reopening policies in Texas, USA. To estimate the model parameters, we used the number of reported confirmed cases and deaths before the reopening policies were implemented, specifically, the data between March 4th (the first documented COVID-19 case in Texas) and April 28th, 2020 (the day right after the announcement of Phase 1 reopening policy). Our model fits the reported data very well (Fig. 2). Based on the estimated model, we simulated the effect of different reopening policies on COVID-19 pandemic in Texas. To our knowledge, this is the first study to investigate the effect of reopening policies in Texas, USA, using a data-driven SEIR transmission model.

According to the estimated SEIR model, if the “stay-at-home order” continued without reopening policy, COVID-19 pandemic could be controlled by the end of 2020 with the lowest number of infected cases, deaths and hospitalizations in Texas. If the reopening policy with strong control and protection measures, i.e., the effective contact rate is low, but the detection rates and quarantine rate were enhanced by 2-folds or higher, the COVID-19 epidemic could be similarly controlled as the case without reopening. However, the data of reported confirmed cases and deaths show that the COVID-19 epidemic in Texas is much worse than these promising cases.

If no any additional control and protection measures could be implemented after reopening, the COVID-19 epidemic would result in a rapid pandemic wave with significantly higher numbers of infected cases, hospitalizations and deaths. Additional control and protection measures with different magnitudes could flatten the epidemic curve and reduce the number of infected cases, hospitalizations and deaths, but the low magnitude of control and protection measures could make COVID-19 last longer. For example, the low-risk reopening with a low magnitude of control measures (the case $S_{12}$ in Table 4) could make the pandemic last for four years (up to March 2024). In this case, the COVID-19 epidemic curve could be flattened with a reduced number of infections, but the effect of COVID-19 epidemic on economy would also last longer.

Compared the simulation results with the reported COVID-19 epidemic data up to July 2020 in Texas, USA, the real-world epidemic pattern is between the cases of the low and high magnitude of control measures (S_22_ and S_23_) with a medium risk of contact rate after reopening (see Fig. 5, Fig. 6). In this case, the pandemic might last until summer 2021 to February 2022 with a total of 4–10 million infected cases and 20,080–58,604 deaths at the end of epidemic. However, if the COVID-19 epidemic continued in other states of USA and the cross-state transmission could not stopped, this result could be affected and changed. The COVID-19 epidemic trajectories could also be affected by new control policies, vaccines and effective treatments in the future.

In this study, the proposed SEIR model shows goodness-of-fit to the reported COVID-19 data before reopening in April 2020, and captured the epidemic trend of COVID-19 after reopening in Texas. We also recognize some limitations of the proposed model and model assumptions. We did not consider the population migration and movement between states in the model. However, since the outbreak of COVID-19, travel restrictions have been implemented and significant cross-state migration and movement could be ignored reasonably. We also assumed that the patients who were confirmed and quarantined at home or hospitalized did not infect others. Due to the data limitation, we also ignored the COVID-19 patients who were dead at home. In the model, we did not consider some big events of gathering, such as annual rodeo and the ‘black life matters’ parade in big cities, that might have a significant effect on COVID-19 epidemic. Most importantly, the proposed SEIR model suffers the model identifiability problem since the data only for the confirmed infected cases and deaths were available and reliable. The data for the number of hospitalizations and recovered patients were available, but were estimated and not reliable. To alleviate this problem, we fixed some of the model parameters based on the literature and used strong constraints for some of the parameters in the parameter estimation. To valid the simulation results in terms of the choices of the fixed parameters and initial values for model fitting, intensive sensitivity analyses were performed. From Figures S3–S10 in the supplemental material we observed that the prediction results are robust to different choices of the fixed parameters and initial values.

This study aims to extend the SEIR model to assess the effect of reopening policies on the COVID-19 epidemic, instead of for accurate predictions of epidemics. The established model can be used to simulate the consequences of different scenarios for different policy changes, which could be used as an evidence-driven guidance for decision-makers to assess the trade-off among different policies. Although our model was developed based on the COVID-19 data of Texas, it could be easily adapted and generalized to other states of USA and other countries.

## Declaration of competing interest

No potential conflict of interest was reported by the authors.
