# Supplementary material for: Assessing effects of reopening policies on COVID-19 pandemic in Texas with a data-driven transmission model
Source: Infect Dis Model. 2021 Feb 23;6:461–73. doi: 10.1016/j.idm.2021.02.001 (PMC7901308; doi:10.1016/j.idm.2021.02.001)
Supplement: Multimedia component 1 [file mmc1.docx]

**SUPPLEMENTAL MATERIAL**

Assessing Effects of Reopening Policies on COVID-19 Pandemic in Texas with a Data-Driven Transmission Model

**Supplemental Figures**

[Figure S1. Predicted daily number of new confirmed cases, deaths, infected cases and hospitalized cases under the high-risk reopening policies 2.](#_Toc55284836)

[Figure S2. Predicted cumulative number of new confirmed cases, deaths, infected cases and hospitalized cases under the high-risk reopening policies 3.](#_Toc55284837)

[Figure S3. Histogram of the model fitting residuals given different fixed parameter values 4.](#_Toc55284838)

[Figure S4. The effect of different fixed parameter values on the predicted number of infections 5.](#_Toc55284839)

[Figure S5. The effect of different fixed parameter values on the predicted number of deaths 6.](#_Toc55284840)

[Figure S6. The effect of different fixed parameter values on the predicted number of hospitalizations 7.](#_Toc55284841)

[Figure S7. Histogram of the model fitting residuals given different initial values 8.](#_Toc55284842)

[Figure S8. The effect of different initial values on the predicted number of infections 9.](#_Toc55284843)

[Figure S9. The effect of different initial values on the predicted number of deaths 10.](#_Toc55284844)

[Figure S10. The effect of different initial values on the predicted number of hospitalizations 11.](#_Toc55284845)


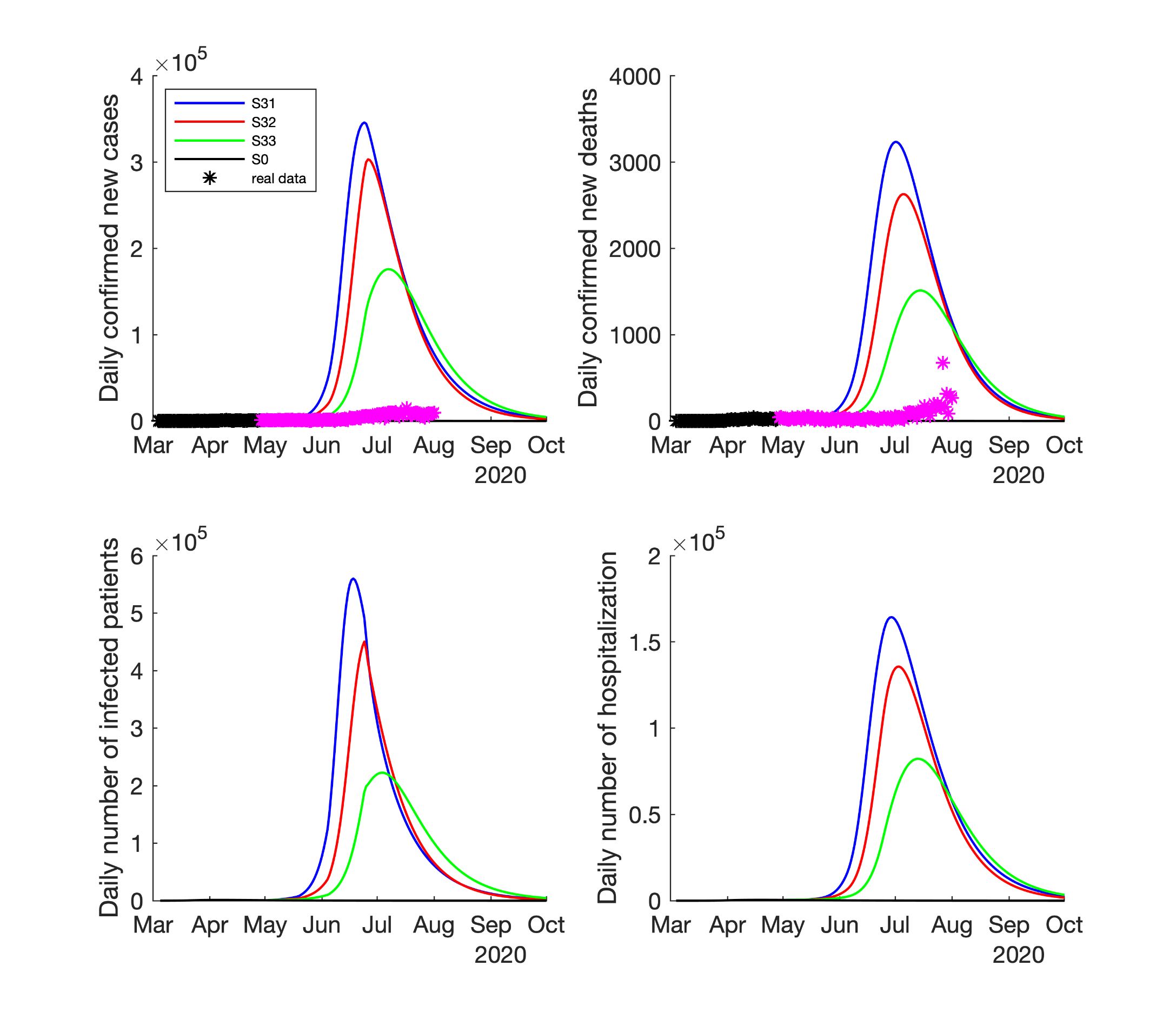


Figure S1. Predicted daily number of new confirmed cases, deaths, infected cases and hospitalized cases if the high-risk reopening policy was implemented, i.e., the contact rate increased by 4 times on May 1^st^, increased by 5 times on May 18^th^, increased by 6 times on June 3^rd^, and reduce to 5 times after June 25^th^. The time span is between March 4^th^ and October 1^st^, 2020. The black * denotes the reported data used for model fitting and purple * denotes the reported data after model fitting.


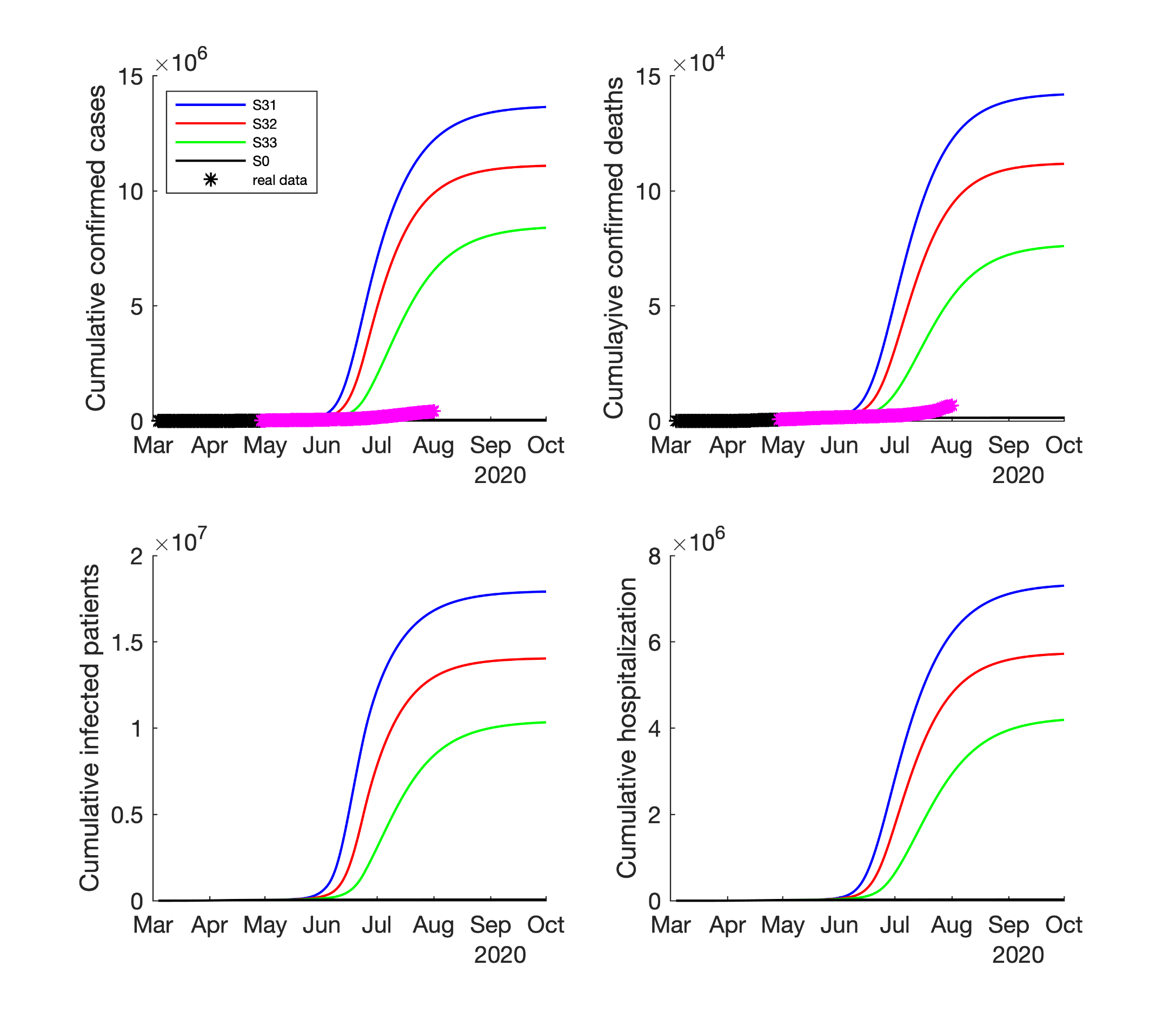


Figure S2. Predicted cumulative number of new confirmed cases, deaths, infected cases and hospitalized cases if the high-risk reopening policy was implemented, i.e., the contact rate increased by 4 times on May 1^st^, increased by 5 times on May 18^th^, increased by 6 times on June 3^rd^, and reduce to 5 times after June 25^th^. The time span is between March 4^th^ and October 1^st^, 2020. The black * denotes the reported data used for model fitting and purple * denotes the reported data after model fitting.


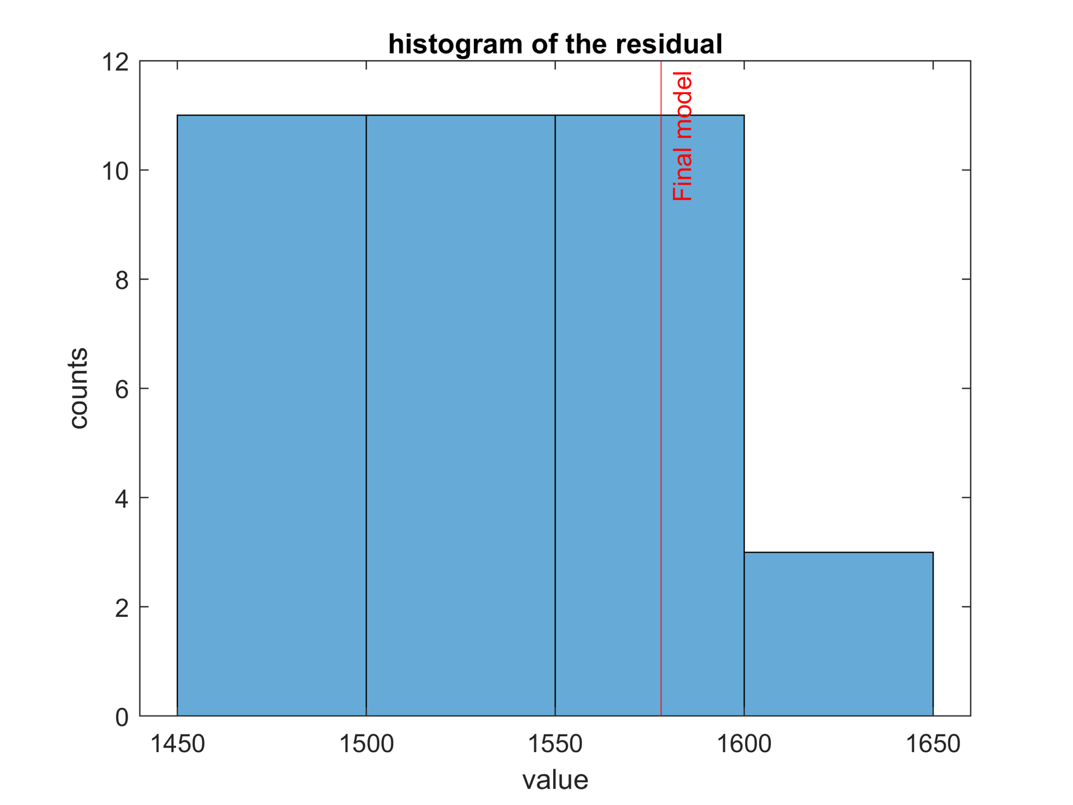


Figure S3. Histogram of the model fitting residuals (objective function) for different fixed parameter values.


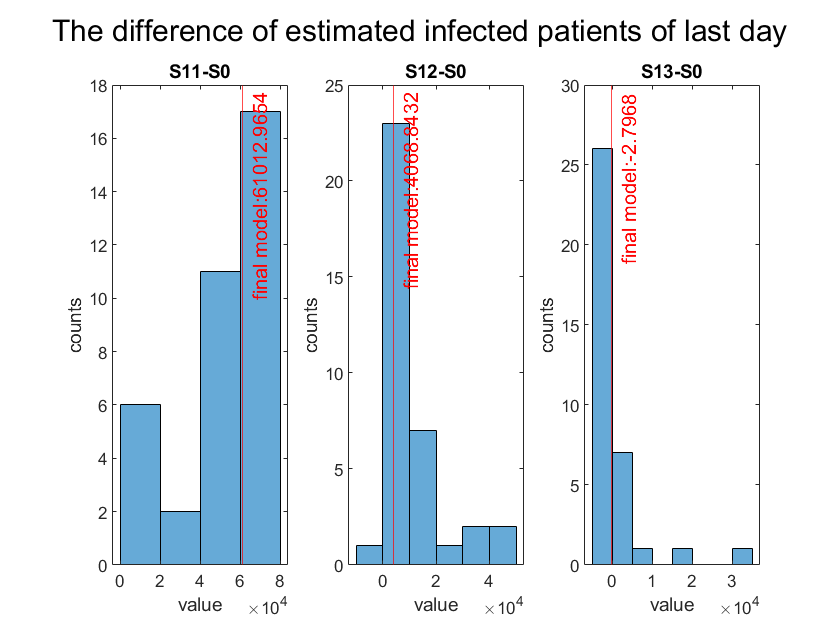


Figure S4. The effect of different fixed parameter values on the prediction of infected people of last day (Oct. 1^st^). The final model refers to our model.


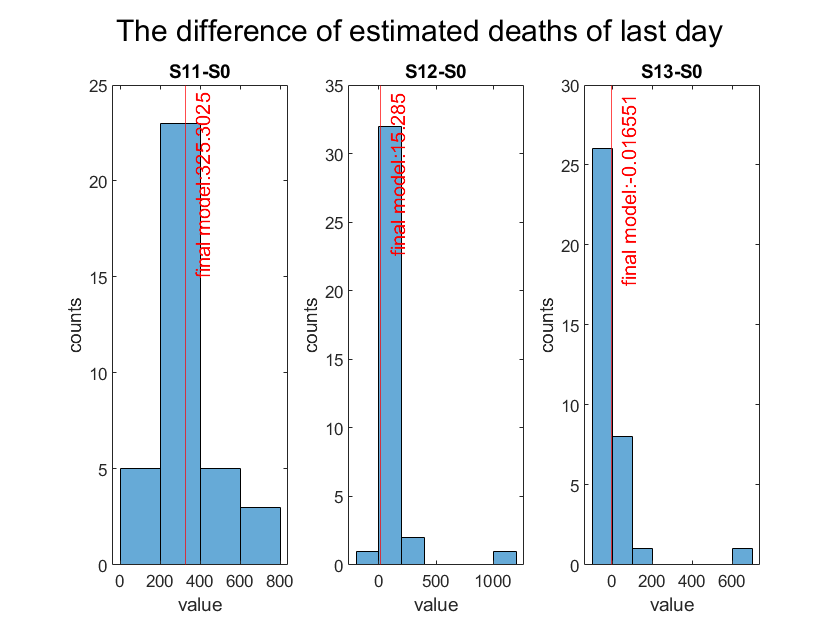


Figure S5. The effect of different fixed parameter values on the prediction of death of last day (Oct. 1^st^). The final model refers to our model.


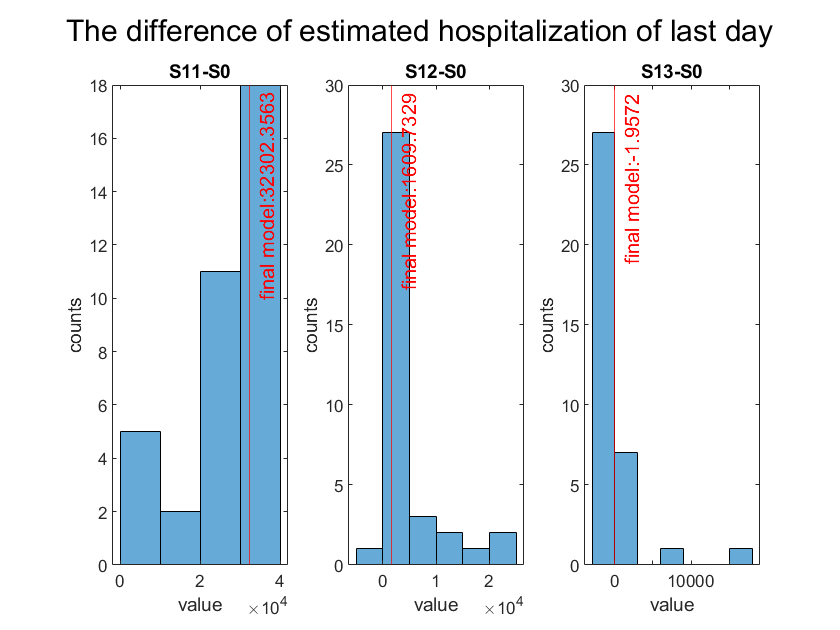


Figure S6. The effect of different fixed parameter values on the prediction of hospitalization of last day (Oct. 1^st^). The final model refers to our model.


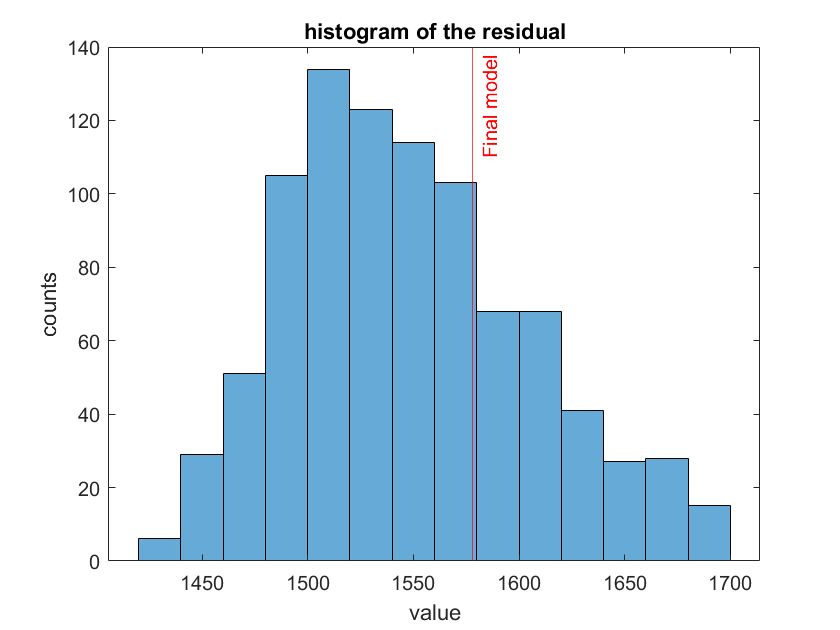


Figure S7. Histogram of the model fitting residuals (objective function) for different initial values.


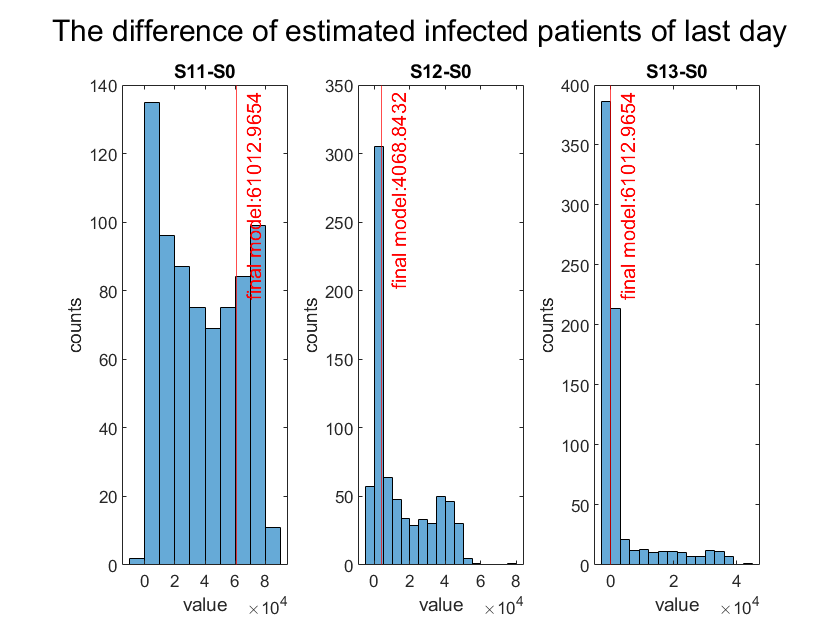


Figure S8. The effect of different initial values on the prediction of infected patients of last day (Oct. 1^st^). The final model refers to our model.


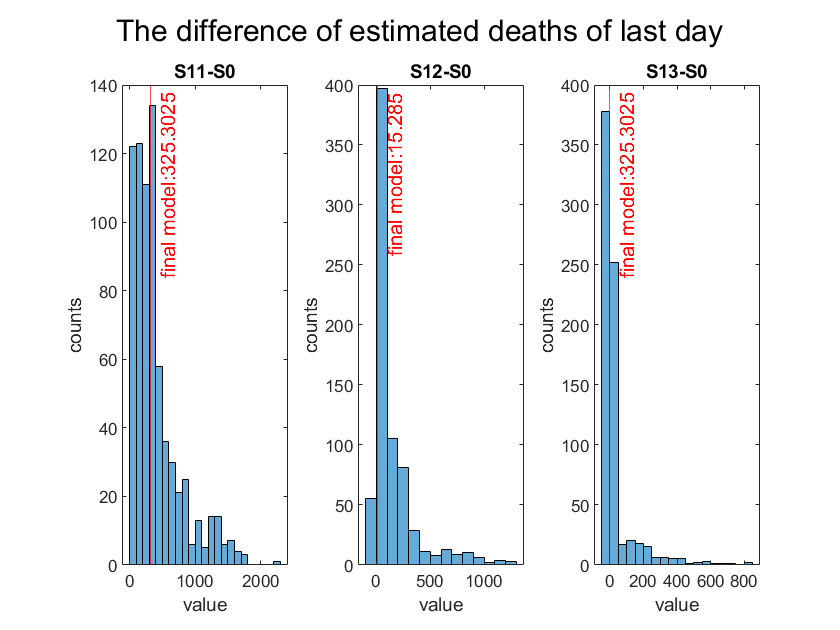


Figure S9. The effect of different initial values on the prediction of death of last day (Oct. 1^st^). The final model refers to our model.


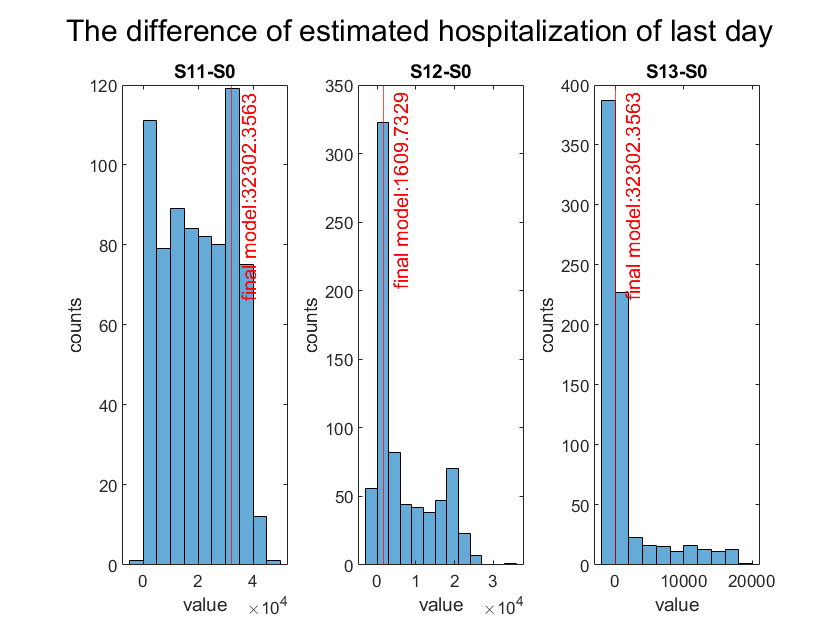


Figure S10. The effect of different initial values on the prediction of hospitalization of last day (Oct. 1^st^). The final model refers to our model.
